# Physicians’ and nurses’ thoughts and concerns about introducing neonatal male circumcision in Thailand: a qualitative study

**DOI:** 10.1186/s12913-018-3093-y

**Published:** 2018-04-11

**Authors:** Kriengkrai Srithanaviboonchai, Namtip Srirak, Boonlure Pruenglampoo, Kanittha Thaikla, Jiraporn Suwanteerangkul, Jiraporn Khorana, Vipa Danthamrongkul, Suchada Paileeklee, Uraiwan Pattanasattayavong, Deanna E. Grimes, Richard M. Grimes

**Affiliations:** 1 Research Institute for Health Sciences, Chiang Mai University, Chiang Mai, Thailand; 2 Faculty of Medicine, Chiang Mai University, Chiang Mai, Thailand; 3 College of Public Health Sciences, Chulalongkorn University, Bangkok, Thailand; 4 Faculty of Medicine, Khon Kaen University, Khon Kaen, Thailand; 5 Faculty of Medicine, Prince of Songkla University, Songkla, Thailand; 6 School of Nursing, University of Texas Health Science Center, Houston, Texas USA; 7 Baylor-UT Houston Center for AIDS Research, Houston, Texas USA; 8 Division of General Internal Medicine, University of Texas Health Science Center at Houston, Houston, Texas USA

**Keywords:** Neonatal circumcision, HIV prevention, Health professional attitudes, Thailand

## Abstract

**Background:**

Neonatal male circumcision (NMC) is an alternative approach to adult male circumcision for HIV prevention. Recent studies found that NMC was rarely performed in Thailand and that most Thai health professionals did not recognize that NMC could reduce the risk of HIV infection and would not want NMC services in their hospitals. This study explored the thoughts and concerns of Thai government health staff regarding the introduction of NMC in government health facilities as a public health measure.

**Methods:**

In-depth interviews with physicians, nurses and physician administrators from four different levels of government hospitals in four provinces representing 4 regions of Thailand were conducted after provision of education regarding the benefits and risks of NMC. Interviews were audio recorded and analyzed using Atlas.ti software to develop themes.

**Results:**

Six themes emerged from the data of 42 respondents: understanding of the benefits of NMC; risks of NMC; need for a pilot project; need for staff training and hospital readiness; need for parental/family education; and need for public awareness educational campaign. Major concerns included possible medical complications of NMC, infringement of child rights, and lack of understanding from staff and parents. The respondents emphasized the need for a clear policy, proper training of staff, financial and equipment support, and piloting NMC rollout before this measure could be fully implemented.

**Conclusions:**

Thai health professionals who took part in this study expressed several concerns if NMC had to be performed in their health care facilities. There is significant preparation that needs to be done before NMC can be introduced in the country.

**Electronic supplementary material:**

The online version of this article (10.1186/s12913-018-3093-y) contains supplementary material, which is available to authorized users.

## Background

It is well established that male circumcision (MC) reduces the risk of female to male transmission of HIV infection. Randomized trials have shown that circumcised men are 50–61% less likely to acquire HIV infection than men who are uncircumcised [1–3]. MC also reduces the chances that women will contract HIV infection because of the lower probability that their partners are infected [4]. As a onetime intervention, MC does not require consistent adherence to preventive behaviors such as condom use or pill taking. As a result, the World Health Organization recommends that countries include MC as part of a comprehensive HIV prevention strategy [5]. Additional health benefits to men from MC include reduced risk of acquiring genital ulcer disease [6], herpes simplex virus and human papilloma virus [7]. which is an important risk factor for penile cancer [8]. MC also benefits female partners of the circumcised men by lowering the risk of women acquiring human papilloma virus, which causes cervical cancer, genital ulcer disease, bacterial vaginosis, and trichomoniasis [9].

Unfortunately, MC is not considered desirable by all boys and young men and acceptance rates range from 29% in Uganda to 87% in Swaziland of surveyed African men [10]. In addition to attitudinal difficulties there are potential medical problems with MC. Studies have shown that there can be significant complications in up to 18% among participants aged 5 to 21 years old even when the procedure is performed in clinical settings [11].

Neonatal male circumcision (NMC) is an alternative to adult male circumcision as an HIV prevention strategy. Recruitment of patients is easier as most babies are born in health care settings. When NMC is performed in hospital settings, complication rates range from 0.3% to 2.4% in developing countries [12]. Healing in infants is rapid, usually taking 7 days [13], while healing in adults may take as long as several weeks [14], and in contrast to adult men, babies are not vulnerable to acquiring sexually transmitted infections, including HIV, during the healing process.

NMC has the potential to become a highly effective long-range strategy for HIV prevention in countries where there is a significant HIV prevalence, a strong health infrastructure, an informed population and supportive health personnel. Thailand is arguably an appropriate country to utilize NMC as HIV prevention measure [12]. The country has the highest prevalence of HIV infection in Asia [15]. It has a well-developed health care system, which is widely accessible for its citizens, and almost all births occur in hospitals [16]. Thailand has some experience with circumcision as Muslim boys are circumcised in a pre-adolescent religious ritual. In addition, for-profit hospitals that cater to a foreign and wealthy clientele provide NMC [17]. However, the majority (~ 90%) Buddhist population does not practice either MC or child circumcision. So, one would presume that the general population is not familiar with the benefits or complications of NMC.

This also seems to be the case with health professionals. A nationwide survey of clinical providers in 562 hospitals with obstetrical services found that the majority of government staff viewed NMC as unsafe, difficult to perform and did not want the service to be implemented in their facilities nor want to be trained [17]. This suggested a need to conduct an in-depth exploration of the knowledge and attitudes of Thai physicians and nurses to better understand the reasons for negative attitudes towards NMC. Therefore, this study explored the thoughts and concerns of health staff regarding the introduction of NMC in government health facilities as a public health measure.

## Methods

This qualitative study was a sub-study of a larger quantitative survey. Detailed procedures for how hospitals were selected were described in another published article [18]. The strategy was to conduct interviews at geographically dispersed governmental hospitals that represented the range of service levels. Four provinces from northern (Chiang Mai), northeastern (Khon Kaen, central (Pathumthani) and southern Thailand (Songkhla) were selected based on the presence of a medical school in the province. This allowed for the selection of a teaching hospital in each region. Each province in Thailand has one provincial hospital which was also included in the study. Two community hospitals were selected for each province, one suburban and one rural.

A practicing physician, the hospital administrator (also a physician) and a head nurse whose job was relevant to NMC at each hospital were purposively selected and asked to participate in-depth interviews. All interviewees had participated in the quantitative portion of the study prior to the interview. They had completed a questionnaire about their knowledge of NMC and had received a one-page information sheet describing the benefits and risks of NMC.

The interviews were conducted in Thai following the interview guide and focused on their concerns about NMC as well as their understanding of its benefits. The interview guide in English is available as a Additional file 1. The respondents also were asked to provide thoughts and advice on how it might be possible to implement NMC in Thailand. The conversations were audio-recorded and transcribed. The data were computerized and analyzed using the Atlas.ti software in order to code and categorize the data and summarize the results into themes. Comments reflecting the thematic results were translated into English for the purposes of this article.

Three institutional review boards (IRB) approved the study. These included the IRBs of: Research Institute for Health Sciences, Chiang Mai University; Faculty of Medicine, Khon Kaen University; and Faculty of Medicine, Chiang Mai University. Verbal informed consent was obtained prior to the interview. All IRBs allowed for verbal informed consent because of the non-sensitive nature of the qualitative study.

## Results

Forty-two of the targeted 48 health care providers were interviewed. Physician involvement from the Southern region was less than planned with only one practicing physician from the four participating hospitals available at the time of the interviews. The distribution of participating personnel by province and professional role can be seen in Table 1. The responses could be categorized into six themes: 1) understanding of the benefits of NMC, 2) concerns about the risks of NMC, 3) need for a pilot project, 4) need for staff training and hospital NMC readiness, 5) need for parental/family education, and 6) need for and NMC public awareness campaign.

**Table 1:** Number and percentage of responding health care personnel by province, and professional role

Province	Administrative Physician (n)	(%)	Practicing Physician (n)	(%)	Nurse (n)	(%)	Total (n)	%
Pathumthani (central)	4	30.9	4	30.8	4	26.7	12	28.6
Khon Kaen (northeastern)	4	30.9	4	30.8	4	26.7	12	28.6
Chiang Mai (northern)	3	27.3	4	30.8	4	26.7	11	26.2
Songkhla (southern)	3	10.9	1	7.7	3	20.0	7	16.7
Total	14	100.0	13	100.0	15	100.0	42	100.0

### Understanding the benefits of NMC

The respondents were not fully aware of the benefits of NMC, especially the reduced risk of HIV infection, prior to the interviews. Most, however, recognized the hygienic benefits of circumcision. For example, one nurse said:

*It’s good. I let my child be circumcised. It is easy to keep the penis clean. There won’t be phimosis. It will decrease phimosis and urinary tract infection… so I got my child circumcised.*

Some understood that NMC could prevent transmission of sexually transmitted diseases including human papilloma virus which causes cervical cancer. As a nurse said:

*It might be an option for the general population in the long run for prevention of sexually transmitted diseases, which is a problem at present, and it should also be seen as a way to prevent cervical cancer. …*

While a physician stated that:

*“I didn’t know that circumcision could reduce the risk of HIV but I did know that it helps reduce the risk of cervical cancer. I haven’t been following HIV research lately...”*

### Concerns about the risks of NMC

The interviews revealed major concerns on the part of providers about the safety and efficacy of NMC surgery. There was fear of possible medical complications, such as bleeding and infection, during and after NMC. Most participants wanted assurance that the procedure would be carried out by highly skilled and experienced staff.

A nurse stated:

*(I’m) concerned about bleeding, infection and care after surgery. Could it really prevent the disease as they said? Could it prevent cancer and sexually transmitted diseases? Is that true?*

“*I am concerned about the experience and skill of doctors … performing the surgery. Nowadays, there are many newly graduated doctors with a very high turnover rate. I don’t think they have enough experience. People will not have confidence. If something bad happens, they will sue us.”*

Some respondents associated pain during surgery with infringement of child rights and cited this as a major concern.

A nurse stated that

*“Don’t agree, I don’t agree. I feel bad for the infants. It causes them pain and may create long-term bad memories, which we might not know about. It also violates the child’s rights. We don’t know what they would feel in the future”*

### Need for a pilot project

When asked for their thoughts or advice about implementing NMC in their hospital the respondents stated that it would be important to provide the scientific evidence to inform personnel of the benefits of NMC. Participants also suggested strategies to effectively introduce NMC. These included implementation of pilot or demonstration projects, adding NMC as one of the requirements in medical training, acquiring needed instruments, and increasing budgets and staff for the hospitals.

A physician stated that:

*“There must be a pilot test. We then expand from that if it works well. There will be a lot of consequences if all hospitals are forced to do this from the beginning…”*

Another physician was of the opinion:

*..If it were to be done, there should be evidence that it is cost-effective. Have to do a pilot and have to follow up for a long period until the children have grown up then (we) could get clear evidence to determine whether it could prevent transmission of HIV and other diseases. Then it could turn into a policy. Should not follow for only 1-2 years then conclude as a policy.*

An administrator had a similar opinion stating that there should be:

*... a pilot project and have a clear result for the public then follow up for 3-5 years*

A head nurse had a similar opinion:

*If it is government policy, there should be a pilot project in hospitals that are ready to do this. If it’s done in all hospitals at the same time, there may be some unforeseen problems. The pilot hospitals may be the secondary or tertiary hospitals if they are ready. Ready means having enough equipment and personnel so they could run the pilot. If they face problems, they should be solved before launching in all hospitals. There should be criteria for selecting hospitals for the pilot project, and team. The physicians who are responsible for this procedure may be general physicians, not necessary a specialist, since specialists are only in tertiary hospitals.*

### Need for staff training and preparing the hospital for NMC

Most participants specifically stressed the need for proper training of the staff that would perform NMC.

This was stated by an administrator:

*…For me, what I would like to ask for is just training, to review the length of foreskin that we can cut, how to suture without causing much bleeding. Just training for surgical skill.*

A second administrator had a similar concern.

*..If it becomes government policy, the government should provide knowledge to personnel, train nurses in care after surgery, make equipment and surgical rooms available.*

Another administrator seemed to consider that the procedure was so complicated that it needed highly trained physicians to perform the procedure.

*…need support…need personnel…if possible, need specialist physicians who have more experience then general physician such as urologist or pediatric surgeon, and anesthetic nurse in some instances. No need for the surgical room and equipment. We do need the budget for it. If it is policy, the government should provide support for this.*

Physicians had concerns about training but were also worried about making certain that there would be adequate budget and staffing before implementing NMC.

*…If there is circumcision service, the number of surgeons should increase, especially surgeons for newborns. If general physicians are trained, the parents may worry since they are not specialists. This is the biggest worry for parents. We will need more equipment should this procedure be provided free of charge f under the universal health insurance system. The budget should be calculated based on the number of deliveries at the hospital.*

*…if we have to arrange for this service, the following should happen: the physicians should have experiences with the procedure, the hospital should get budgetary support for this procedure from the National Health Security Office (NHSO), should have equipment, and the physicians and nurses should get training.*

### Need for parental/family education

Several physicians commented about the necessity of parental education:

*I worry about the confidence of the family since neonatal circumcision is not a routine procedure for newborns…It would be a big worry, since it formerly was not standard procedure for newborns.*

*Have to talk with all families to help them understand and to prepare the caretakers. They have to sign the consent forms.*

*I worry whether parents will accept this, so should do the procedure only if the families agree to do so…. We may not be able to do circumcisions for all newborns, maybe only those whose family has agreed and given their consent.*

*The parents’ understanding is important…may try to convince those who don’t want circumcision,… may have to have examples of cases to help with their decision.*

### Need for educating the general public about NMC

A number of the practicing physicians commented on the importance of public education:

*…need support for media to educate the public and should have a strategy for public awareness which should be done both individually and through mass media*

…*I think that it needs to be advertised to the public. …We can start in the hospitals that provide delivery services. We can give health education from the start of ANC through birth. If the baby is a boy we can examine whether he has phimosis. If so, we advise about the procedure and the care team. If the baby and family are ready, then they can come for service.*

The campaign must be in the long term and happen at the same time

…*It’s never been part of the culture for newborns so public awareness must be done over a long period. All hospitals should provide the service at the same time, university hospitals and provincial hospital. The public should feel that it’s general practice. Large hospitals should be the model then we can get the reference as the standard*

Similar issues were raised by nurses.

…*should create an understanding among the public so they are confident and cooperate. If not, there may be problems of charging and suing after the service*

*…There must be public awareness to prepare the people. Public health personnel should run a campaign in the hospital, give health education during prenatal care to prepare the parents. It’s similar to promoting family planning or the public awareness campaign from the Ministry for vaccination. It’s the same as promotion of Influenza or SARS via television. Public awareness campaign may have both a positive and negative impact. People may think that it helps to decrease HIV (infection) so they may take risks. So, the information provided for the public should focus on cervical cancer prevention which benefits both women and men.*

An administrator stated that:

*“This is possible if there is a campaign throughout the country. But it needs preparation and understanding, which is difficult. … It’s not our tradition. Even sterilizing women to prevent pregnancy took long time to be accepted….”*

## Discussion

The study showed that health professionals were willing to participate in a study that explored the attitudes and knowledge about NMC. They were willing to make suggestions about the practical issues that would need to be considered before implementing a national NMC program. They also brought up themes that could provide the basis for quantitative studies in other places. While there is significant potential benefit to implementing NMC in Thailand, a great deal of preparation will be necessary before launching a program to implement NMC.

From the respondents’ responses, there seems to be significant lack of knowledge that MC could help reduce the risk of HIV acquisition. This will need to be alleviated before there will be any likelihood of successful implementation of NMC in the country. Increasing teaching about the solid evidence on this issue is warranted in both medical schools, nursing schools and in-service trainings. This education should make the case that NMC is preferable to MC performed at a later age because recovery is faster, there are fewer complications, and the importance of circumcising before exposure to HIV, HPV, etc. This suggests that medical staff in obstetrics and in pediatrics should also be the primary focus of educational campaigns. A vaccine that had 50% efficacy would be hailed as a breakthrough and widely adopted, which has not been the case with MC.

Medical staff who participated in the study were not well informed about the low levels of risk of NMC. They expressed their concerns about the safety of the intervention and were skeptical with regard to the scientific evidence in favor of NMC. Emphasizing information about the low rate of complications is warranted both in schools and in-service training. The participants also thought that NMC is difficult to perform and required a specialist physician, anesthesia, special equipment, etc. In places where NMC is widely practiced it is frequently performed by non-surgeons without general anesthesia and requires simple, disposable equipment. There also was concern about the post-operative care of the infant although in countries where NMC is routine practice, simple instructions are given to mothers and most of the care is done at home after discharge. Medical staff need to see that the procedure is simple and can be carried out by general practitioners and that there are devices that accomplish circumcision safely with minimal bleeding or need for follow-up. Nurses should be a particular target of these information efforts since they will likely be the interface with the family. Medical students and physicians will need training in how to perform the procedure and nurses will require training in post-operative care of the infants and in teaching mothers how to provide home care.

The respondents recognized the importance of educating the general public about the utility of NMC. They acknowledge that this should be done through mass media for the general public and with one-on-one education with parents throughout the antenatal period. Evidence from Africa is that mothers, once informed of the benefits, often support MC. This means that health professionals need to be educated about both the advantages of NMC and how to counsel parents who are contemplating whether their infant son should have the procedure since educating the public will only be effective if medical staff are also supportive of NMC. The health education program will help people have a better understanding of NMC, have more confidence in its safety and effectiveness, and gain the cooperation of parents and the public. This health education campaign should occur at the same time as when hospitals begin to offer NMC. The campaign will have to be conducted over a number of years until NMC becomes commonplace. The content should include a focus on risk reduction to diseases other than HIV infection such as cancer so that it is understood that NMC benefits both men and women.

An interesting finding is that the interviewees regularly cited the need for pilot projects to determine the feasibility of implementing NMC on a national scale. The suggestions that there be small scale experiments in real settings is cogent and should be strongly considered as Thailand moves forward with investigating this disease preventing procedure. Pilot projects focused on how to deliver the message about NMC would also be valuable given the current lack of knowledge about the procedure.

There were some limitations regarding this study. The small and purposive samples of each category of health professionals might not represent and reflect the thoughts of everyone. Most participants just knew the benefits and risks of NMC just prior to the interviews from our one-page information sheet. This might be too little information and too brief time for them to provide us with thoughtful comments. This kind of study is also local-context specific. Its results may not be generalized to other countries.

## Conclusions

Most Thai health professionals who took part in this study did not recognize the benefit of NMC on HIV prevention prior to the interview. They expressed several concerns about performing NMC in their health care facilities and suggested preparation that is needed before NMC can be introduced in the country.

## Additional file

Additional file 1: In-depth interview guide. Interview guide for the interviewers to in-depth inter interview the personnel of the hospitals. (DOCX 13 kb)

## Abbreviations

IRB: Institutional review board
MC: Male circumcision
NMC: Neonatal male circumcision
