# Supplementary material for: Physicians’ and nurses’ thoughts and concerns about introducing neonatal male circumcision in Thailand: a qualitative study
Source: BMC Health Serv Res. 2018 Apr 11;18:275. doi: 10.1186/s12913-018-3093-y (PMC5896126; doi:10.1186/s12913-018-3093-y)
Supplement: Supplementary file 1 — In-depth interview guide. Interview guide for the interviewers to in-depth inter interview the personnel of the hospitals. (DOCX 13 kb) [file 12913_2018_3093_MOESM1_ESM.docx]

**Interview guide for in-depth inter interviews the personnel of the hospital.**

**For the director of the hospital.**

- What do you think about circumcision?
- Do you know about the results of research, providing circumcision reduces risk of HIV infection or not?
- How do you think if the ministry of public health to promote newborn male circumcision in government hospitals?
- What kind of supports you need if the newborn male circumcision to be serviced in your hospital.
- How would you arrange the newborn male circumcision in your hospital?
- What are your concern or worry if you need to provide neonatal male circumcision in your hospital?

**For head nurse**

- What do you think about circumcision?
- Do you know about the results of research, providing circumcision reduces risk of HIV infection or not?
- How do you think if the ministry of public health to promote newborn male circumcision in government hospitals?
- What kind of supports you need if the newborn male circumcision to be serviced in your hospital.
- How would you arrange the newborn male circumcision in your hospital?
- What are your concern or worry if you need to provide neonatal male circumcision in your hospital?

**For doctor**

- Have you performed circumcision?
- What do you think about circumcision?
- Do you know about the results of research, providing circumcision reduces risk of HIV infection or not?
- How do you think if the ministry of public health to promote newborn male circumcision in government hospitals?
- What kind of supports you need if the newborn male circumcision to be serviced in your hospital.
- How would you arrange the newborn male circumcision in your hospital?
- Which surgical procedure and equipment should be used?
- What are your concern or worry if you need to provide neonatal male circumcision in your hospital?
